# Cytotoxic Activity of Crude Extracts as well as of Pure Components from *Jatropha* Species, Plants Used Extensively in African Traditional Medicine

**DOI:** 10.1155/2011/134954

**Published:** 2011-06-15

**Authors:** Olapeju O. Aiyelaagbe, Amao A. Hamid, Ernesto Fattorusso, Orazio Taglialatela-Scafati, Heinz C. Schröder, Werner E. G. Müller

**Affiliations:** ^1^Department of Chemistry, University of Ibadan, Ibadan 200284, Nigeria; ^2^Department of Chemistry, University of Ilorin, Ilorin 24003, Nigeria; ^3^Dipartimento di Chimica delle Sostanze Naturali, Università di Napoli Federico II, Via D. Montesano, 49, 80131 Napoli, Italy; ^4^Institute for Physiological Chemistry, University Medical Center of the Johannes Gutenberg University Mainz, Duesbergweg 6, 55128 Mainz, Germany

## Abstract

Extracts from *Jatropha curcas*, a plant used in African traditional medicine for various diseases, were tested for cytotoxic activity. The root extracts strongly reduced cell growth of tumor cells *in vitro*, a result consistent with the knowledge of the application of these plant extracts in traditional medicine, especially to cure/ameliorate cancer. A selection of pure diterpenoids existing in extracts from *Jatropha* species and isolated from *J. curcas*, for example, curcusone C, curcusone D, multidione, 15-epi-4Z-jatrogrossidentadion, 4Z-jatrogrossidentadion, 4E-jatrogrossidentadion, 2-hydroxyisojatrogrossidion, and 2-epi-hydroxyisojatrogrossidion, were likewise tested, and they also showed strong cytotoxic activity. It turned out that these extracts are highly active against L5178y mouse lymphoma cells and HeLa human cervix carcinoma cells, while they cause none or only very low activity against neuronal cell, for example, PC12. These data underscore that extracts from *J. curcas* or pure secondary metabolites from the plant are promising candidates to be anticancer drug, combined with low neuroactive effects.

## 1. Introduction

**Figure 1:**
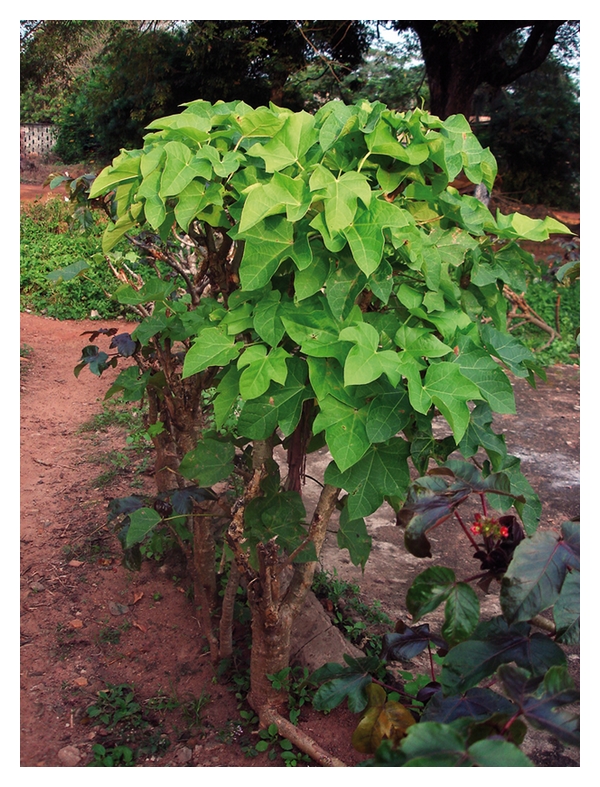
The plant *Jatropha curcas*, in the local language also termed “lapalapa funfun”, is used in the South Western and Middle Belt regions (Nigeria) as live fences. Extracts from the root and rootbarks are used for medicinal applications.

**Figure 2:**
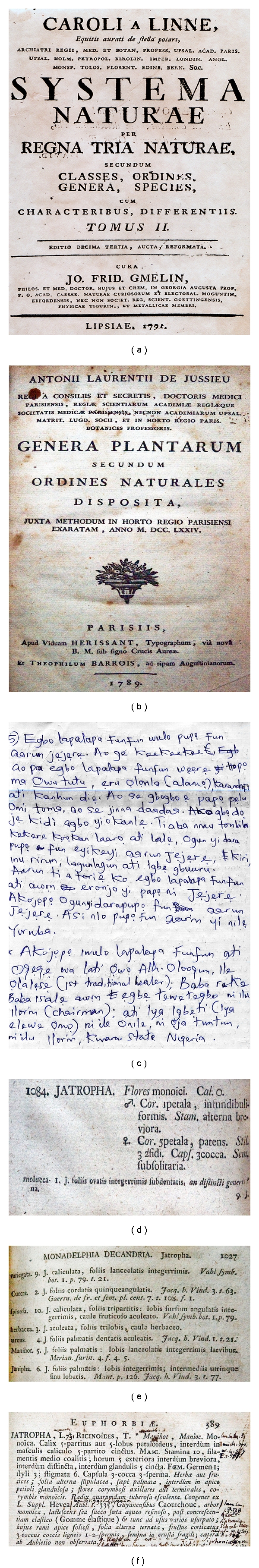
The first descriptions of *J. curcas*. (a) Description of the species by von Linné [3]. (b) First description of *J. curcas* as a medical plant by de Jussieu [5]. (c) Recipe describing *J. curcas* extracts as antitumor medicine form the traditional healer, Baba Reke. (d, e) Description of the genus Jatropha by von Linné [3, 4], already including 13 species (f).

*Jatropha curcas* is a multipurpose (both medicinal and biotechnologically important) plant and drought resistant large shrub which is widely cultivated in Africa and Asia for aesthetics as a live fence (Figure 1), as a source for the production of biodiesel and for medicinal purposes (reviewed in [1]). The plant belongs to the genus *Jatropha* and family Euphorbiaceae, known for their toxic constituents [2]. While the seeds are used for biodiesel production, the root and rootbark are, among the other parts of the plant, of medicinal relevance. The first description of *J. curcas* was published by von Linné [3] who gave a few years later a more comprehensive description of the genus *Jatropha* including more species [4] Figures 2(a), 2(d), and 2(e). de Jussieu [5] was the first to describe the medical use of extracts from *J. curcas* as being useful if given orally (tunc ad usus varios usurpato) (Figures 2(b) and 2(f)).

In traditional medicine, this plant has been applied since the earliest times for the cure of various ailments ranging from simple fevers to infectious diseases including sexually transmitted diseases in many African and Asian countries [6, 7]. The different parts of the plant serve various medicinal uses. The seeds and the seed oil are used as purgative and as a remedy for syphilis. In addition, the oil is used as a substitute for diesel oil and as fuel [8, 9]. The leaves are applied extensively in West African ethnomedical practice in different forms to cure various ailments like fever, mouth infections, jaundice, guinea worm sores, and joint rheumatism [10, 11]. The sap (latex) and crushed leaves have also shown antiparasitic activity [12, 13]. Furthermore, the latex contains compounds displaying antibacterial activity against *Staphylococcus aureus* [14]. Water extract of the branches strongly inhibited HIV- induced cytopathic effects with low cytotoxicity [15]. In addition, the stems contain compounds with strong antimicrobial activities, as proven by studies carried out in Nigeria, and are used as chewing sticks in parts of that country [16, 17]. Extracts of the stems have been suggested to possess various biological activities including anti-insect, antimicrobial, cytotoxic, anti-inflammatory, and molluscicidal activities [18–25]. The roots of *J. curcas* are used after decoction as a mouthwash for bleeding gums, toothache, eczema, ringworm, and scabies, and to cure dysentery and venereal diseases, like gonorrhea [6, 11]. 

Previous phytochemical studies on *J. curcas* resulted in the isolation of many compounds including diterpenes, sterols, flavonoids, alkaloids, and peptides [28–34]. Many of these compounds have shown various biological activities ranging from antimicrobial to anticancer. Curcusone A and B isolated from the stems showed anticancer activities while curcusone B, furthermore, effectively suppressed the metastatic processes at nontoxic doses, [35]. It should be stressed, however, that the anticancer activities of curcusone C and D have not been reported previously.

In this paper, we report the cytotoxic/cytostatic activities of root extracts and chemical constituents of *Jatropha* species against certain cancer cell lines along with evidence from traditional healers on the use of this plant in treating different types of cancer. In contrast to the studies published to date, in which pure compounds had been used for the cytotoxicity studies, for example, curcin [36], we analyzed extracts from the plant *J. curcas*, similar to those that are used for treatment in traditional medicine in the South West and Middle belt regions of Nigeria. The data show that both the extracts from *J. curcas* as well as its diterpenes are highly active against tumor cells, L5178y mouse lymphoma cells, and HeLa human cervix carcinoma cells, combined with a low effect on the neuronal cell line PC12.

## 2. Materials and Methods

### 2.1. Plant Materials

Whole plants of *Jatropha curcas* (Euphorbiaceae) were collected from the campus of the University of Ibadan, Ibadan, Nigeria in 2006. The plants were authenticated at the Herbarium of the Forestry Research Institute, Ibadan, Nigeria. (Herbarium Voucher Number: FHI 107674). The roots were separated from the rest of the plant, dried, and ground.

### 2.2. Extraction of Plant Materials

A known amount of the dried, ground root of *J. curcas* (1.0 kg) was extracted with n-hexane, ethyl acetate, and methanol to give the respective crude extracts which were concentrated *in vacuo*. The yields of the extracts were as follows: hexane 10.5 g, ethyl acetate 11.2 g, and methanol 42.3 g.

### 2.3. Fractionation of the Extracts and Isolation of the Pure Compounds

Based on the results of the *in-vitro* cytotoxicity assay of the extracts, the hexane and ethyl acetate extracts *J. curcas* were subjected to fractionation by column chromatography. The fractions obtained were subjected to further assays before they were purified by HPLC to obtain the pure compounds. Details of the procedures and characterisation of the compounds have been reported [37]. Fourteen compounds were isolated from the extracts, and some of these compounds had been isolated from *Jatropha podagrica *and* Jatropha multifida *(Euphorbiaceae), medicinal plants closely related to *J. curcas* and which also grows in Nigeria [21, 22, 38, 39].

### 2.4. Confirmation from the Herbalists on the Efficacy of *J. curcas* as Anticancer Agents

**Figure 3:**
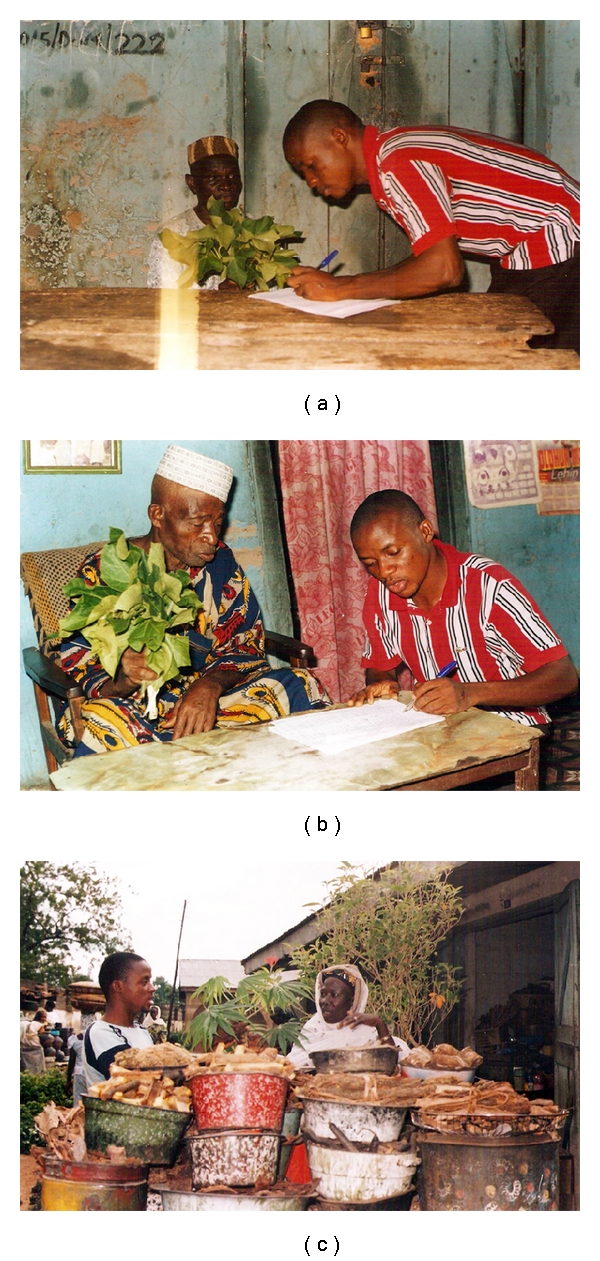
Medical impact of extracts from *J. curcas*, as learned from interviews with (a) the traditional healer, Alhaji Oloogun, Ilorin (Kwara state, Nigeria), (b) Baba Reke (Ilorin), and (c) Iya Igbeti, a female Yoruba traditional pediatric pharmacist at the New Market at Ilorin. They described how extracts of this plant, supplemented with black pepper or local black soap (as prepared by the Yoruba tribes of Nigeria), are used as traditional medicine which may also increase appetite and promote secretion, properties which have also been documented in modern medicinal textbooks [50].

Two traditional healers and one traditional pediatric pharmacist (herbal seller) were thoroughly interviewed to collect more detailed information on the uses and efficacies of the plant in traditional medicine. The interviews were conducted orally in the vernacular of the people, and the responses were transcribed on paper and subsequently translated by an anthropologist for accuracy. They gave useful information on the different applications involving *J. curcas* to treat various diseases including cancer (Figures 2 and 3).

### 2.5. Determination of Cytotoxic Activity

The extracts obtained were subjected to an *in-vitro* cytotoxicity assay applying the MTT [3-(4,5-dimethylthiazol-2-yl)-2,5-diphenyl tetrazolium bromide] (Sigma-Aldrich, Taufkirchen; Germany) method [39–41]. Three different cell lines were routinely used for activity testing: L5178y mouse lymphoma cells [42], PC12 rat adrenal medulla pheochromocytoma cells [43], and also HeLa human cervix carcinoma cells [44]. The cells were grown in RPMI 1640 medium (Gibco BRL, Eggenstein; Germany), supplemented with 10 mM Hepes [hydroxyethyl-piperazineethane-sulfonic acid], 10% fetal calf serum (FCS) (PAA, Cölbe, Germany) for L5178y and HeLa cells, or 5% horse serum for PC12 cells, and 0.1% gentamycin. All cells were routinely passaged twice weekly. The cells were kept in a humidified atmosphere of 95% air and 5% CO_2_ at 37°C.

### 2.6. MTT Assay

To estimate the EC_50_ values, L5178y, PC12, or HeLa cells were incubated for 72 hrs in the presence of different concentrations (0.1; 0.3; 1.0; 3.0, and 10.0 *μ*g/mL) of the respective extracts. The final volumes in the assays were 200 *μ*L. All extracts were dissolved in DMSO [dimethyl sulfoxide] (stock solution 10 mg/mL) and stored at −20°C. The viability of the cells was determined using the MTT colorimetric assay system. The evaluation was performed in 96-well plates at 595 nm using an ELISA plate reader, after overnight incubation at 37°C as described [45].

### 2.7. Statistics

The 50% effective concentration (EC_50_), representing that concentration at which the growth rate of the infected cells was reduced by 50%, was estimated by logit regression [26], as described [27]. The means (±SD) from 10 separate experiments are given.

## 3. Results and Discussion

### 3.1. Biomedical Application in Traditional Medicine in the South West/Middle Belt Part of Nigeria

Over 30 different traditional healers and herbal sellers have been interviewed. The interviews were conducted orally in the vernacular of the people, and their responses were transcribed on paper and subsequently translated by an anthropologist for accuracy. The following three representative recipes should be documented here.

The traditional healer Alhaji Oloogun (Figure 3(a)) gave a recipe for the amelioration of skin inflammations (Figure 2(c)) as follows. The roots must be minced and are then extracted together with 1 : 100 (w/w) Yoruba small sized local pepper with aqueous ethanolic solvent. The slurry is thoroughly mixed 1 : 5 (v/v) with local black soap. This formulation is then used for everyday bathing in order to treat skin rashes and infections very effectively.

From the second traditional healer, Baba Reke (Figure 3(b)), a recipe was obtained that has frequently been applied to treat sexually transmitted diseases. The *J. curcas* roots are cut into small pieces and then soaked with an aqueous alcoholic solvent for two to three days. The cleared extract is used as a drink; small glass cups (about 10 mL each) of this extract must be drunk twice daily, morning and night. By the experience of this healer, this formulation was successful against any kind of cancer and several types of sexually transmitted diseases, for example, gonorrhea.

Finally, from Iya Igbeti, a female Yoruba traditional pediatric pharmacist (Figure 3(c)), a recipe for the treatment/amelioration of a broad spectrum of local diseases was obtained. The aqueous extract is again prepared from *J. curcas* roots, together with the following components; first, with the dried outer parts of the *Cassia tora* fruits, also termed “eru olounla” or “alamo,” and, second, with dried stem bark of guinea corn, *Sorghum caudatum*, “karandafi” by using small quantity of each (1 : 10 w/w). In order to increase the efficiency of the extraction, the slurry is supplemented with potash (1 : 10 v/w) and natural cotton wool (1 : 10 v/w). The suspension is then boiled at 70°C (60 min) and subsequently heated at 100°C for another 60 min after addition of an equal volume of clean water until it is well cooked. Care should be taken not to allow the extract to rapidly chill. The patients are advised to drink a small glass cup of that formulation twice daily in the morning and night. It was found to be effective against cancer of the intestine, stomach upset, and rheumatism as well as diarrhea.

### 3.2. Cytostatic Activity of the Extracts

**Figure 4:**
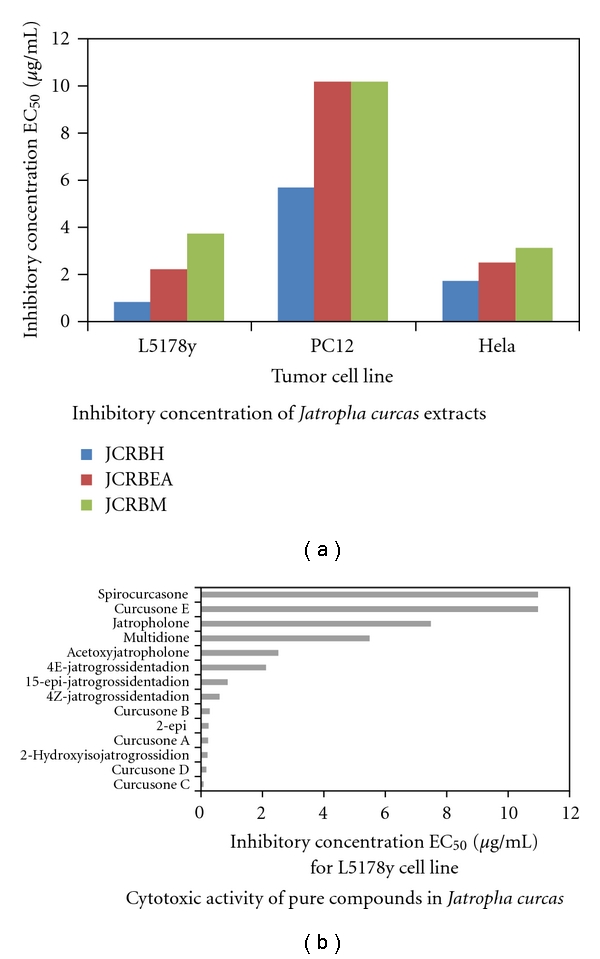
(a) Cytotoxic activity of *J. curcas* extracts. Further details are given in Table 1. (b) Cytotoxic activity of pure compounds from extracts of *J. curcas*. The pure compounds had been isolated from root extracts as described under Section 2. The means of 10 different experiments (±SD) are given as EC_50_: effective doses; see also Table 2 for further information.

The extracts obtained showed strong cytotoxic activity against the three cell lines with effective inhibition higher than 100% even at concentrations below 5 *μ*g/mL. This concentration had been set as a borderline, below which a compound/extract is termed cytotoxic [46]. The data summarized in Table 1 and in Figure 4(a) show that the hexane extract (JCRBH) possessed highest inhibitory activity. It is remarkable that besides the strong inhibition of L5178y cells, also HeLa cells were affected by the hexane extract. Most cytostatic drugs are much less inhibitory on HeLa cells than on L5178y cells [45]. The extracts prepared with ethyl acetate and methanol were less effective. Remarkable is the weaker effects of all three extracts, especially of those obtained with the more polar solvents, (ethyl acetate and methanol) on PC12 cells. Since this cell line [47] is of neuronal origin it can be expected that also *in vivo* these extracts might be less/if at all toxic on neuronal tissue.

Previously, some pure compounds have been isolated from organic extracts of *J. curcas* and other *Jatropha* species [30, 38, 48, 49]. Here, we isolated fourteen diterpenes by bioassay-guided techniques as described earlier [37]. These compounds were tested for their inhibitory activities on the L5178y cell line (Table 2; Figure 4(b)). Some of the diterpenes showed very strong inhibitory activities. Especially high was the inhibition for curcusone C with EC_50_ of 0.08 *μ*g/mL. Four curcusones have already earlier been isolated from *J. curcas* out of which two, namely curcusone A and B, have shown cytotoxic activities [35]. The present study is, however, the first report of the cytotoxic activities for curcusone C and D. The highly potent activities exhibited by curcusone C and D suggest that these compounds could be developed further as anticancer drugs. Furthermore, their modes of action have to be investigated; for curcusone B, an antimetastatic activity has already been described [35].

**Figure 5:**
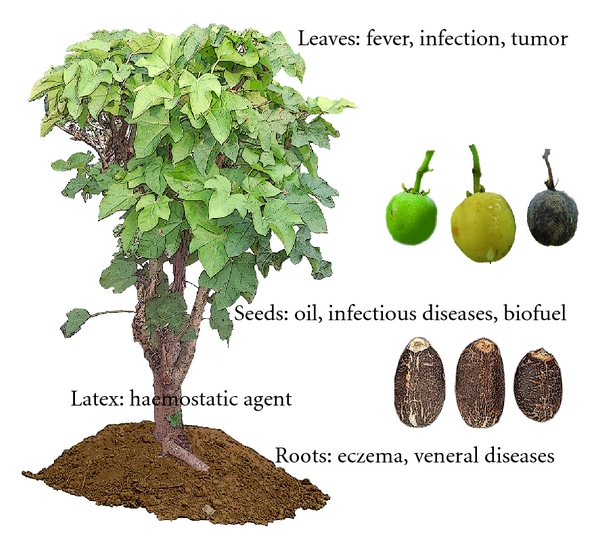
*Jatropha curcas*: from leaves, latex, roots, and seeds different bioactive or commercially important ingredients have been extracted; schematic representation. For details, see text.

In conclusion, the present study underscores that the plant *J. curcas* produces a series of cytostatically active compounds; Figure 5. In view of the present findings that even crude extracts from this plant are highly active, and relatively more active than some of the hitherto known secondary metabolites from this plant, it can be expected that more new compounds with higher cytostatic activity can be isolated in future. However, it should also be considered that the active compounds in the extracts may act synergistically in such a way that the inhibitory effects of the extracts are higher than that of their components. Such a potentiation of individual compounds in extracts, especially from herbs has been discussed before [50, 51]. 

## Figures and Tables

**Table 1:** Percentage growth inhibition by 50% (EC_50_: effective dose, causing 50% reduction of cell density during the 72 hrs incubation) by extracts from *J. curcas* roots. The following extracts were tested: hexane extract (JCRBH) and ethyl acetate extract (JCRBEA) as well as methanol extract (JCRBM). The three cells lines L5178y, PC12, and HeLa were used for the experiments.

Extracts	Tumor cell lines, inhibitory concentration EC_50_ (*μ*g/mL)		
	L5178y	PC12	HeLa
JCRBH	0.82 ± 0.1	5.7 ± 0.6	1.7 ± 0.1
JCRBEA	2.2 ± 0.2	>10.0	2.5 ± 0.3
JCRBM	3.7 ± 0.3	>10.0	3.1 ± 0.3

**Table 2:** Cytotoxic activity of compounds isolated from *Jatropha curcas* root extracts. The EC_50_ values are given here; the SD's of the respective values are less than 15%.

Compounds	EC_50_ (*μ*g/mL) for L5178y cells
Curcusone C	0.08
Curcusone D	0.16
2-Hydroxyisojatrogrossidion	0.20
Curcusone A	0.21
2-epi-hydroxyisojatrogrossidion	0.24
Curcusone B	0.27
4Z-jatrogrossidentadion	0.60
15-epi-4E-jatrogrossidentadion	0.85
4E-jatrogrossidentadion	2.10
Acetoxyjatropholone	2.50
Multidione	5.50
Jatropholone	7.50
Curcusone E	>10
Spirocurcasone	>10
